# QuickStats

**Published:** 2015-07-17

**Authors:** FROM THE NATIONAL CENTER FOR HEALTH STATISTICS

**Figure:**
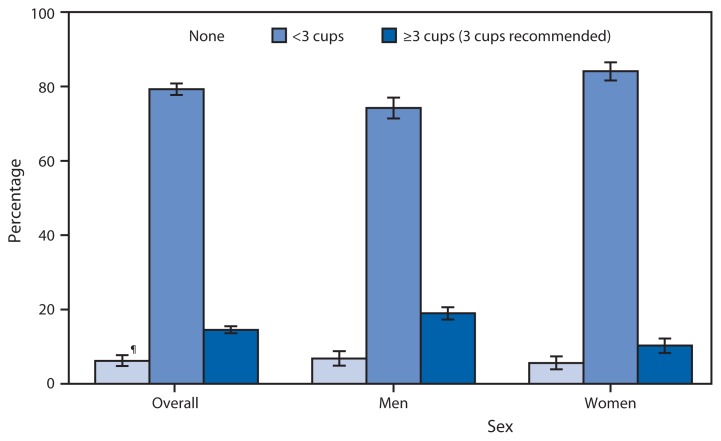
Percentage of Adults Aged ≥20 Years Who Consumed Dairy* on a Given Day,^†^ by Amount^§^ and Sex — National Health and Nutrition Examination Survey, United States, 2011–2012 * Dairy is one of nine “main components” listed in the U.S. Department of Agriculture’s Food Patterns Equivalents Database. Dairy consists of milk, yogurt, and cheese. Multi-ingredient foods containing dairy, such as pizza or ice cream, are also assigned to the dairy component, and cup equivalents are calculated for the individual ingredients. Additional information is available at http://www.ars.usda.gov/SP2UserFiles/Place/80400530/pdf/fped/FPED_1112.pdf. ^†^ The National Health and Nutrition Examination Survey asks participants to name all the foods and beverages they consumed in the preceding 24 hours. Data from the Day 1 24-hour recall were used in this analysis. ^§^ *Dietary Guidelines for Americans*, which is jointly published every 5 years by the U.S. Department of Health and Human Services and the U.S. Department of Agriculture, recommends that adults consume 3 cups of dairy per day. ^¶^ 95% confidence interval.

During 2011–2012, on a given day, 14.5% of adults aged ≥20 years consumed the U.S. recommended 3 cups of dairy. Most adults (79.3%) consumed some dairy (<3 cups), and 6.2% of adults consumed no dairy. More men (19.0%) than women (10.3%) consumed the recommended 3 cups of dairy.

**Source:** CDC. National Health and Nutrition Examination Survey Data. Hyattsville, MD: US Department of Health and Human Services, CDC, National Center for Health Statistics; 2011–2012. Available at http://www.cdc.gov/nchs/nhanes.htm.

**Reported by:** Samara Joy Nielsen, PhD, snielsen@cdc.gov, 301-458-4193; Donna G. Rhodes, MS; Steven M. Frenk, PhD.

